# Thyroid-related hormones as potential markers of hypoxia/ischemia

**DOI:** 10.1007/s13577-020-00341-x

**Published:** 2020-03-07

**Authors:** Naoto Tani, Mayumi Ishikawa, Miho Watanabe, Tomoya Ikeda, Takaki Ishikawa

**Affiliations:** 1 Department of Legal Medicine, Osaka City University Medical School, Asahi-machi 1-4-3, Abeno, Osaka, 545-8585 Japan; 2 Forensic Autopsy Section, Medico-Legal Consultation and Postmortem Investigation Support Center, c/o Department of Legal Medicine, Osaka City University Medical School, Asahi-machi 1-4-3, Abeno, Osaka, 545-8585 Japan; 3 Center of Endocrinology, Diabetes and Arteriosclerosis, Nippon Medical School Musashikosugi Hospital, Kosugi-cho 1-396, Nakahara-ku, Kawasaki, 211-8533 Japan; 4 Laboratory of Clinical Regenerative Medicine, Laboratory of Advanced Research, Department of Neurosurgery, Faculty of Medicine, University of Tsukuba, D326 1-1-1, Tennodai, Tsukuba, Ibaraki 305-8575 Japan

**Keywords:** Asphyxia, Ischemia/hypoxia, Thyroid hormone, Thyroid-stimulating hormone, Cultured cell

## Abstract

This study aimed to investigate the usefulness of the thyroid-related hormones as markers of acute systemic hypoxia/ischemia to identify deaths caused by asphyxiation due to neck compression in human autopsy cases. The following deaths from pathophysiological conditions were examined: mechanical asphyxia and acute/subacute blunt head injury; acute/subacute non-head blunt injury; sharp instrument injury as the hemorrhagic shock condition; drowning as alveolar injury; burn; and death due to cardiac dysfunction. Blood samples were collected from the left and right cardiac chambers and iliac veins, and serum triiodothyronine (T3), thyroxine (T4), thyroglobulin (Tg), and thyroid-stimulating hormone (TSH) levels were measured using electrochemiluminescence immunoassays. Two types of thyroid cell lines were used to confirm independent thyroid function under the condition of hypoxia (3% O_2_). The human thyroid carcinoma cell line (HOTHC) cell line derived from human anaplastic thyroid carcinoma and the UD-PTC (sample of the second resection papillary thyroid carcinoma) cell line derived from human thyroid papillary adenoma, which forms Tg retention follicles, were used to examine the secretion levels of T3, T4, and Tg hormones. The results showed a strong correlation between T3 and T4 levels in all blood sampling sites, while the TSH and Tg levels were not correlated with the other markers. Serum T3 and T4 levels were higher in cases of mechanical asphyxia and acute/subacute blunt head injury, representing hypoxic and ischemic conditions of the brain as compared to those in other causes of death. In the thyroid gland cell line, T4, T3, and Tg levels were stimulated after exposure to hypoxia for 10–30 min. These findings suggest that systemic advanced hypoxia/ischemia may cause a rapid and TSH-independent release of T3 and T4 thyroid hormones in autopsy cases. These findings demonstrate that increased thyroid-related hormone (T3 and T4) levels in the pathophysiological field may indicate systemic hypoxia/ischemia.

**Electronic supplementary material:**

The online version of this article (10.1007/s13577-020-00341-x) contains supplementary material, which is available to authorized users.

## Introduction

During pathological autopsies, it is very important to distinguish deaths due to acute systemic hypoxia/ischemia caused by asphyxiation due to neck compression from those due to sudden natural causes (e.g., acute ischemic heart disease) [1, 2]. Previous studies examining this issue from the perspective of forensic pathology, biochemistry, and molecular biology [3–5] suggested that thyroid hormones could be used as markers of direct physical thyroid gland stimulation following cervical compression [6, 7]. A previous post-mortem blood analysis showed that, although the thyroglobulin (Tg) and triiodothyronine (T3) levels increased with cervical compression, those of thyroxine (T4) and thyroid-stimulating hormone (TSH) remained within the normal range, indicating that these changes were most likely caused by the agonal stage or post-mortem changes, rather than hormonal regulation via the hypothalamic–pituitary–thyroid axis [7]. Moreover, another study reported increasing Tg levels and decreasing TSH levels associated with head trauma unrelated to asphyxiation, suggesting that these changes could be attributed to abnormal secretion rather than hypothalamic control [8]. Therefore, there is still a lack of consensus regarding the evidence on whether changes in thyroid-related hormone levels can be considered accurate markers of asphyxiation due to neck compression. 

Thyroid-related hormone secretion is triggered by the release of TSH from the adenohypophysis. This TSH release initially promotes the reabsorption of Tg accumulated in the thyroid follicles by follicular epithelial cells and is subsequently digested by intracellular lysosomes to release extrafollicular T3 or T4 that circulates through the blood capillaries. Although T3 accounts for approximately 2% of the total thyroid hormones in the blood, it exerts a substantially greater physiological activity than T4 [9]. In vivo investigations have reported that, although hypoxia at high altitudes does not induce changes in TSH levels, the serum levels of T3 and T4 increase [10, 11]. Furthermore, studies using rat models of hypoxia exposure reported an increase in the levels of type 3 iodothyronine deiodinase, which converts T4 to the inactive 3,3,5′-triiodo-l-thyronine [12, 13]. However, very little is known about the pituitary–thyroid endocrinological changes that accompany hypoxic ischemia in humans. Therefore, the present study investigated changes in thyroid-related hormone levels in cases of hypoxic ischemia by biochemical analysis.

Additionally, in the present study, a hypoxic experiment using a thyroid hormone-secreting HOTHC-sc-4D7 sub-clone derived from human anaplastic thyroid carcinoma (HOTHC) and a UD-PTC cell line derived from human thyroid papillary adenoma was conducted to corroborate the biochemical data from the autopsy materials. The HOTHC-sc-4D7 cells do not form follicles and the UD-PTC cell line forms thyroid follicles with the same thyroid hormone secretion pattern as in vivo [14, 15]. The presence or absence of follicular formation is expected to result in different thyroid hormone secretion patterns. Therefore, there was a possibility of peculiar reactions in the HOTHC-sc-4D7 cell line, so that the UD-PTC cell line was also used for confirmation. The mechanism driving the changes in concentrations of thyroid-related hormones under hypoxic conditions was also examined using autopsy materials and these thyroid gland cell lines in vitro.

## Materials and methods

### Autopsy samples

This study included a total of 96 serial forensic autopsy cases, 64 men and 32 women (median age at death: 67 years; range: 25–96 years), that were within 12–72 h post-mortem. The inclusion criteria were cases that had been witnessed and/or for which there was well-established circumstantial evidence to confirm survival and the post-mortem period estimated from classical pathological findings [16]. A complete autopsy and macromorphological, micropathological, and toxicological examinations identified the following causes of death: asphyxia (total *n* = 21; of which, hanging: *n* = 8, strangulation: *n* = 6, and other: *n* = 7) and blunt injury (*n* = 11); acute/subacute non-head injury (*n* = 6); sharp instrument injury as the hemorrhagic shock condition (*n* = 6); drowning as pulmonary alveolar destruction, excluding incidents in the bathtub (*n* = 9); and death due to cardiac dysfunction (*n* = 6). Burns (*n* = 37) were sub-divided into cases with low (< 30%, *n* = 10), intermediate (30–60%, *n* = 16), and high (> 60%, *n* = 11) carboxyhemoglobin (CO-Hb) levels. Patients showing thyroid gland diseases were excluded from this study and the sample demographics are shown in Table 1. Clearly verifiable cases with well-established circumstantial evidence and no significant complications were included in all groups.

**Table 1:** Case profiles for the medicolegal autopsy cases included in this study (*n* = 96)

Cause of death	*n*	Male/female	Age (years)		Survival time	Post-mortem time (h)	
			Range	Median		Range	Median
Asphyxia							
Hanging	8	5/3	26–70	51.5	Acute	12–60	36.0
Strangulation	6	3/3	27–87	57.0	Acute	24–48	36.0
Other^a^	7	4/3	30–81	72.0	Acute	12–48	24.0
Blunt injury							
Head injury							
Acute	7	5/2	25–83	52.0	Acute	12–48	36.0
Subacute	4	4/0	47–72	58.5	Subacute	24–36	30.0
Non-head injury							
Acute	3	1/2	66–70	69.0	Acute	24–48	48.0
Subacute	3	3/0	44–65	65.0	Subacute	12–36	24.0
Sharp instrument injury	6	5/1	45–86	66.5	Acute	24–36	36.0
Drowning	9	4/5	33–96	73.0	Acute	12–48	24.0
Fire fatalities							
CO-Hb < 30%	10	7/4	41–86	78.5	Acute	12–72	24.0
CO-Hb = 30–60%	16	11/5	50–84	71.0	Acute	12–48	24.0
CO-Hb > 60%	11	7/4	28–91	71.0	Acute	12–60	24.0
Cardiac dysfunction	6	6/0	40–75	66.0	Acute	12–60	36.0
Total	96	64/32	25–96	67.0	−	12–72	24.0

^a^Other; choking (*n* = 3), traumatic asphyxia (*n* = 3), smothering (*n* = 1)

### Biochemical analyses

Blood samples were collected aseptically from the left and right heart chambers and the external iliac veins using syringes, centrifuged immediately to separate the sera, and then stored at −  20 °C until use. Serum T3, T4, Tg, and TSH levels were measured using electrochemiluminescence immunoassays [17, 18] and the measurement ranges and sensitivities were: 0.005–100 and 0.002 μIU/ml for TSH; 0.20–6.51 and 0.03 ng/ml for T3; 0.42–24.9 and 0.04 μg/dl for T4; and 0.1–1000 and 0.2–0.5 ng/ml for Tg, respectively. The accuracy, reliability, and reproducibility of the results were checked using serial tenfold dilutions and non-reproducible data were excluded from subsequent analyses. The clinical serum reference intervals for the hormones were as follows: TSH 0.500–5.00 μIU/ml; T3 0.8–1.60 ng/ml; T4 6.10–12.4 μg/dl; and Tg  <  33.7 ng/ml.

### Histopathological analysis

We performed histopathological analysis of the brain in all cases of mechanical asphyxia. Since the hippocampus is known to be particularly susceptible to the effects of ischemia/hypoxia, we evaluated area CA4 of the hippocampus of all specimens. For this, 4 μm thick, routine hematoxylin–eosin (HE)-stained sections of the hippocampus were prepared.

### Toxicological analyses

The blood CO-Hb saturation (%) levels were analyzed using a CO-oximeter system (Ciba-Corning 270; Corning Corp., Corning, NY, USA) [19, 20], while the blood cyanide and alcohol levels were determined using head space gas chromatography/mass spectrometry [21]. Drug analyses were performed using gas chromatography/mass spectrometry [22].

### Cell culture and thyroid hormone measurements

#### Single-cell cloning method for establishment of a HOTHC-sc-4D7 sub-clone from the HOTHC cell line

This experiment used a hormone-secreting HOTHC-sc-4D7 sub-clone derived from the human anaplastic thyroid carcinoma (HOTHC) cell line (obtained from the Clinical Regenerative Medicine, Department of Neurosurgery, University of Tsukuba). The HOTHC cell line was established from an anaplastic carcinoma of the thyroid. One of the thyroid hormones, T3, is secreted by HOTHC cells in small amounts. However, immunocytochemical staining showed that a few cells were stained strongly with anti-T3 antibody. Then, the aim was to achieve a high level of T3-secreting cells by a single-cell cloning method. A single HOTHC cell/ml in the culture medium (GM) solution was prepared and 200 µl of GM solution was poured into each well of a 96-well plate. Wells containing a single cell were marked and single cells were cultured and then 12 subclones of HOTHC cells were established. One of the sub-clones, HOTHC-sc-4D7, which produced the largest amount of T3, was used in the present study [14]. GM, consisting of Dulbecco’s Modified Eagle Medium: Nutrient Mixture F-12 (DMEM/F12) with 10% fetal bovine serum (FBS) (F7524; Sigma-Aldrich, St Louis, MO, USA) with no antibiotics, was incubated with or without HOTHC-sc-4D7 cells at 37 °C. The amount of hormones secreted by the HOTHC-sc-4D7 cells in the conditioned media, except for the amount of these hormones in GM incubated under the same conditions, was then measured. The cells were either cultured in hypoxic conditions, defined as a humidified atmosphere containing 4.7% CO_2_ and 3% O_2_ at 37 °C [23, 24], or normoxic conditions, defined as humidified atmosphere containing 4.7% CO_2_ and 20% O_2_. Since humans are known to die when oxygen levels decreased to 2–5% and 3% O_2_ has been reported to cause functional neuronal cell damage, the experiment was conducted under conditions of 3% O_2_ [25]. The cell counts, measured using a Bürker–Türk counting chamber [26] and a dye exclusion test [27], were adjusted to 1.4 × 10^6^ cells/ml. The doubling time of HOTHC-sc-4D7 cells was 32 h. 

For immunocytochemical staining, the cultured cells were plated at a density of 1 × 10^5^ cells per well in four-well chamber slides (IWAKI, Tokyo, Japan). The cells were fixed with 99.8% methanol for 15 min at − 30 °C (Wako Pure Chemical Industries) and incubated in Blocking One Histo (Nacalai Tesque, Kyoto, Japan) for 10 min at room temperature. Thereafter, the cells were incubated with primary antibodies against T3 and T4 (supplied by the Laboratory of Clinical regenerative Medicine, Department of Neurosurgery, Faculty of Medicine, University of Tsukuba) overnight at 4 °C, followed by Alexa Fluor 488-conjugated goat anti-rabbit IgG secondary antibodies (1:1000 dilution; Thermo Fisher Scientific Inc., Waltham, MA, USA) for 30 min in the dark at room temperature. The nuclei were stained using VECTASHIELD^®^ HardSetTM mounting medium containing DAPI (Vector Laboratories, Burlingame, CA, USA) and images were obtained using a confocal laser scanning microscope (LSM-510; Carl Zeiss, Jena, Germany). The negative control was incubated with Alexa Fluor 488-conjugated goat anti-rabbit IgG without a primary antibody overnight at 4 °C [28]. Furthermore, absorption tests were performed using T3 (5 μg/ml, T2877; Sigma-Aldrich) and T4 (1 μg/ml, T2376; Sigma-Aldrich) and the primary antibodies were used thereafter.

Perfusion culture was carried out to measure hormone secretion over a short period of time and the T3, T4, and Tg levels in the culture fluid were subsequently measured using an enzyme-linked immunosorbent assay (ELISA). The amount of T3 in the conditioned medium was measured by a T3 Accubind ELISA Kit (125-300A; MonoBind Inc., CA, USA), the amount of T4 was measured by a T4 Accubind ELISA Kit (225-300A; MonoBind Inc.), and the amount of Tg was measured by a Tg ELISA Kit (2225-300A; MonoBind Inc.). Each culture fluid was collected for 5 min before each assay point. To confirm cell activity, the levels of vascular endothelial growth factor (VEGF) in the culture fluid were measured using a solid-phase enzyme immunoassay, since HOTHC-sc-4D7 cells are known to express large amounts of it in compared to other cultured cells [29].

#### Sample of second resection papillary thyroid carcinoma (UD-PTC)

The UD-PTC cell line derived from human papillary adenocarcinoma of the thyroid [15] was used. When the cells of this cell line are cultivated (static culture), floating balloons are easily formed. These balloons are similar to the follicles of the thyroid. The UD-PTC cells secreted thyroid hormones. These balloons were put into Rose’s chamber and cultivated by a perfusion culture apparatus using DMEM/F12 supplemented with 10% FBS. The amount of T3 in the conditioned medium was measured by a T3 ELISA Kit (ab108685; Abcam, Tokyo, Japan), the amount of T4 was measured by a T4 ELISA Kit (ab108661; Abcam), and the amount of Tg was measured by a Tg ELISA Kit (ab155441; Abcam). Each culture fluid was collected for 5 min before each assay point. Only for the assay of Tg, these balloons were destroyed by vigorous pipetting in the medium, and then Tg in the medium was assayed. For hypoxic incubation, the perfusion culture apparatus was put into the incubator with 3% O_2_, 4.7% CO_2_, and 92.3% N_2_. For normoxic incubation, 4.7% CO_2_ and 95.3% air were used. To confirm cell function in UD-PTC cells under the hypoxic condition, levels of hypoxia-inducible factor-1 alpha (HIF-1α) and VEGF were measured. The amount of HIF-1α was measured by a RayBio^®^ Human HIF-1 alpha ELISA Kit (ELH-HIF1a-1; RayBiotech, Inc., GA, USA), and VEGF was measured by a Human VEGF ELISA Kit (ab100662; Abcam).

### Statistical analyses

Spearman’s rank correlation coefficient was used to compare pairs of values for the thyroid-related hormone levels. The non-parametric Mann–Whitney *U* test was used for between-group comparisons, while the Games–Howell test was used for analyses involving multiple comparisons. The data are presented as box plots and the maximum serum T3, T4, Tg, and TSH concentrations were log-transformed for graphical presentation only.

Diagnostic relevance was estimated based on the calculated sensitivity, specificity, and accuracy values (i.e., the proportion of correctly predicted cases), and the usefulness of serum T3, T4, Tg, and TSH levels for differentiating between deaths due to asphyxia, blunt head injury, and other causes of death was evaluated using receiver operating characteristic (ROC) curves and the areas under the curves [30]. Youden’s index (sensitivity + specificity − 1) was used to determine the best cutoff values. The results are presented as median and interquartile ranges, unless otherwise stated, and a *p *value of < 0.05 was considered significant. All statistical procedures, including the ROC analyses, were performed using the SPSS 9.0 statistical package (SPSS Inc., Chicago, IL, USA) [31].

## Results

### Correlations between thyroid-related hormone concentrations

Strong correlations were observed between the T3 and T4 concentrations in blood samples from the right cardiac chamber (*r* = 0.535, *p* < 0.001), left cardiac chamber (*r* = 0.575, *p* < 0.001), and iliac vein (*r* = 0.658, *p* < 0.001), and slight correlations were observed between the T3 and Tg levels in samples from the right cardiac chamber (*r* = 0.259, *p* < 0.01) and iliac vein (*r* = 0.218, *p* < 0.05). The T4 and TSH levels in samples from the right (*r* = 0.233, *p* < 0.05) and left (*r* = 0.276, *p* < 0.01) cardiac chambers were also slightly correlated. Although the levels of T3 tended to be higher than those of T4 at multiple sampling sites, no correlations were observed between the T3 and TSH levels across sites (*p* > 0.05) (Fig. S1–3).

### Correlations between hormone levels and causes of death

#### Triiodothyronine (T3)

The levels of T3 were higher in blood samples from cases of asphyxia (right cardiac chamber, median 5.89 ng/ml; left cardiac chamber, median 3.25 ng/ml; iliac vein, median 2.67 ng/ml) than those from cases with other causes of death (right cardiac chamber, median 2.44 ng/ml; left cardiac chamber, median 1.32 ng/ml; iliac vein, median 1.26 ng/ml) except for acute/subacute blunt head injury (right cardiac chamber, median 4.08 ng/ml; left cardiac chamber, median 1.85 ng/ml; iliac vein, median 1.55 ng/ml), irrespective of the sampling site (Table S1). Exceptions included levels in right heart blood in cases of fire fatalities (CO-Hb > 60%) and sudden cardiac death, those in the left cardiac chamber in cases of drowning and sudden cardiac death, and levels in the iliac vein in cases of fire fatalities (CO-Hb < 30%).

**Fig. 1:**
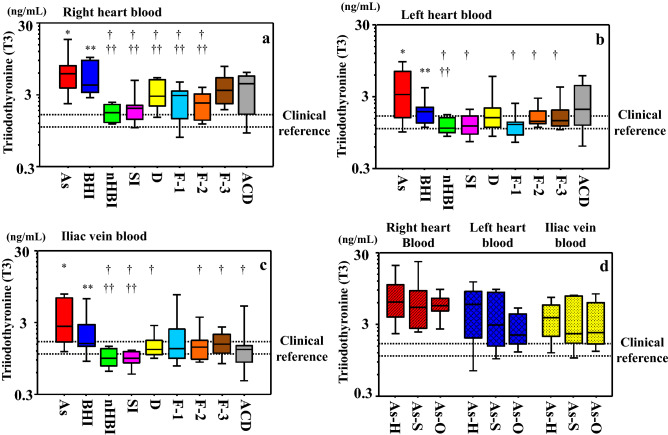
Serum triiodothyronine (T3) concentrations in samples collected from the **a** right cardiac chamber, **b** left cardiac chamber, and **c** iliac vein by cause of death. T3 levels were significantly higher in asphyxia than other cause of death, and (* vs †; *p* < 0.05–0.001), than the blunt head injury cases show significantly higher levels of T3 than in some other cause of death (** vs ††; *p* < 0.05). **d** T3 concentrations by asphyxia subtype (hanging, strangulation, and other). No significant differences are observed. The line bisecting each box represents the median, while the continuous lines outside each box represent the 90% confidence intervals. Concentrations are log-transformed for graphical presentation only. The range between the two dotted lines in the graph indicates the clinical reference value. *As* asphyxia, *BHI* blunt head injury, *nHBI* non-head blunt injury, *SI* sharp instrument injury, *D* drowning, *F-1* fire fatality (CO-Hb < 30%), *F-2* fire fatality (CO-Hb = 30–60%), *F-3* fire fatality (CO-Hb > 60%), *ACD* acute cardiac death

Acute/subacute blunt head injury cases showed significantly higher median levels of T3 than those in the right cardiac chamber in cases of non-head blunt injury, sharp instrument injury, drowning and fire fatality (CO-Hb < 30% and CO-Hb = 30–60%), in the left cardiac chamber in cases of non-head blunt injury, and in the iliac vein in non-head blunt injury and sharp instrument injury (*p* < 0.05–0.0001; Fig. 1a–c). Within the asphyxia group, no differences by subtype (hanging and strangulation as neck compression, and others; Fig. 1d) were observed between sampling sites. Cutoff values of 3.11, 1.62, and 1.49 ng/ml were calculated for distinguishing higher and lower T3 levels (asphyxia and acute/subacute head injury vs. other groups) in samples from the right cardiac chamber (sensitivity 0.84; specificity 0.64), left cardiac chamber (sensitivity 0.69; specificity 0.69), and iliac vein (sensitivity 0.78; specificity 0.69), respectively.

#### Thyroxine (T4)

Similarly, higher T4 levels were observed in samples from cases of asphyxia (right cardiac chamber, median 10.4 µg/dl; left cardiac chamber, median 7.93 µg/dl; iliac vein, median 9.78 µg/dl) compared to those from cases of other causes of death (right cardiac chamber, median 6.92 µg/dl; left cardiac chamber, median 6.23 µg/dl; iliac vein, median 5.72 µg/dl) except for acute/subacute blunt head injury (right cardiac chamber, median 7.65 µg/dl; left cardiac chamber, median 6.58 µg/dl; iliac vein, median 6.97 µg/dl), irrespective of the sampling site (*p* < 0.05–0.001) (Table S1). Exceptions included samples of right and left cardiac blood in cases of fire fatalities (CO-Hb = 30–60%, and CO-Hb > 60%) and sudden cardiac death, and those of iliac vein blood in fire fatalities (CO-Hb < 30%) and sudden cardiac death.

**Fig. 2:**
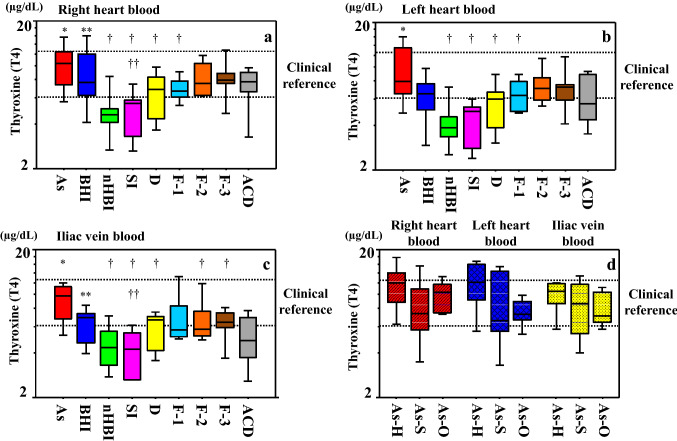
Serum thyroxine (T4) concentrations in samples collected from the **a** right cardiac chamber, **b** left cardiac chamber, and **c** iliac vein by cause of death. T3 levels were significantly higher following asphyxia than with almost all other causes of death (* vs †; *p* < 0.05–0.001). Blunt head injury cases showed significantly higher levels of T3 than cases of sharp instrument injury, except for blood samples from the left cardiac chamber (** vs ††; *p* < 0.05). **d** T4 levels by asphyxia subtype (hanging, strangulation, and other). No significant differences were observed. The line bisecting each box represents the median, while the continuous lines outside each box represent the 90% confidence intervals. The area between the two dotted lines in the graph indicates the clinical reference value. Concentrations are log-transformed for graphical presentation only. *As* asphyxia, *BHI* blunt head injury, *nHBI* non-head blunt injury, *SI* sharp instrument injury, *D* drowning, *F-1* fire fatality (CO-Hb < 30%), *F-2* fire fatality (CO-Hb = 30–60%), *F-3* fire fatality (CO-Hb > 60%), *ACD* acute cardiac death

Blood samples from cases of acute/subacute blunt head injury demonstrated significantly higher median T4 levels (right cardiac chamber, 7.65 µg/dl; left cardiac chamber, 6.58 µg/dl; iliac vein, 6.97 µg/dl) than right cardiac chamber and iliac vein samples from patients with sharp instrument injury (*p* < 0.05; Fig. 2a–c). Within the asphyxia group, no significant differences by subtype (hanging and strangulation as neck compression, and others; Fig. 2d) were observed between sampling sites. Estimated cutoff values of 7.37, 6.50, and 6.91 µg/dl were calculated to distinguish higher and lower T4 levels (asphyxia and acute/subacute head injury vs. other groups) in samples collected from the right chamber (sensitivity 0.78; specificity 0.56), left cardiac chamber (sensitivity 0.72; specificity 0.56), and iliac vein (sensitivity 0.69; specificity 0.72), respectively.

#### Thyroglobulin (Tg)

**Fig. 3:**
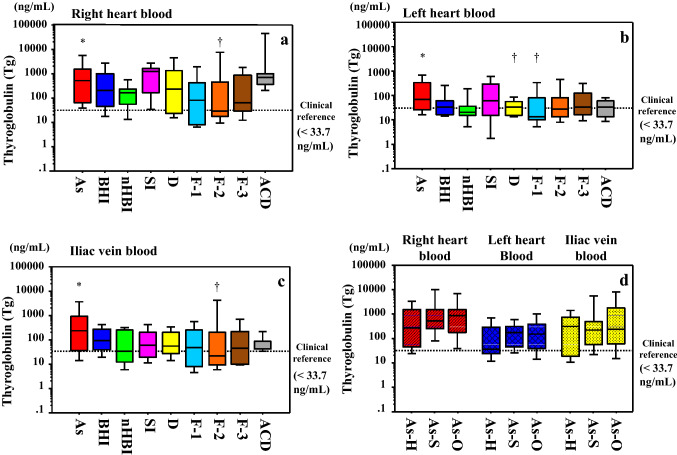
Serum thyroglobulin (Tg) concentrations in samples collected from the **a** right cardiac chamber, **b** left cardiac chamber, and **c** iliac vein, according to the cause of death. Asphyxia cases showed significantly higher levels of Tg than cases of other causes of death (* vs †; *p* < 0.05). **d** Tg concentrations by asphyxia subtype (hanging, strangulation, and other). No significant differences are observed. The line bisecting each box represents the median, while the continuous lines outside each box represent the 90% confidence interval. The area below the dotted line in the graph indicates the clinical reference value (< 33.7 mg/mL). Concentrations are log-transformed for graphical presentation only. *As* asphyxia, *BHI* blunt head injury, *nHBI* non-head blunt injury, *SI* sharp instrument injury, *D* drowning,* F-1* fire fatality (CO-Hb < 30%), *F-2* fire fatality (CO-Hb = 30–60%), *F-3* fire fatality (CO-Hb > 60%), *ACD* acute cardiac death

Higher Tg levels were observed in cases of asphyxia compared to cases of fire fatality in the right cardiac chamber and iliac vein (CO-Hb = 30–60%), and in the left cardiac chamber in cases of fire fatality (CO-Hb < 30%) and drowning (*p* < 0.05). No other significant differences were observed (Fig. 3a–c) (Table S2).

Within the asphyxia group, no differences in Tg levels by subtype (hanging and strangulation as neck compression, and others; Fig. 3d) were observed between the sampling sites. Estimated cutoff levels of 244.0, 35.9, and 107.5 ng/ml were calculated to distinguish higher and lower Tg levels (asphyxia vs. non-head blunt injury) in samples from the right cardiac chamber (sensitivity 0.67; specificity 0.59), left cardiac chamber (sensitivity 0.67; specificity 0.59), and iliac vein (sensitivity 0.62; specificity 0.68), respectively.

#### Thyroid-stimulating hormone (TSH)

**Fig. 4:**
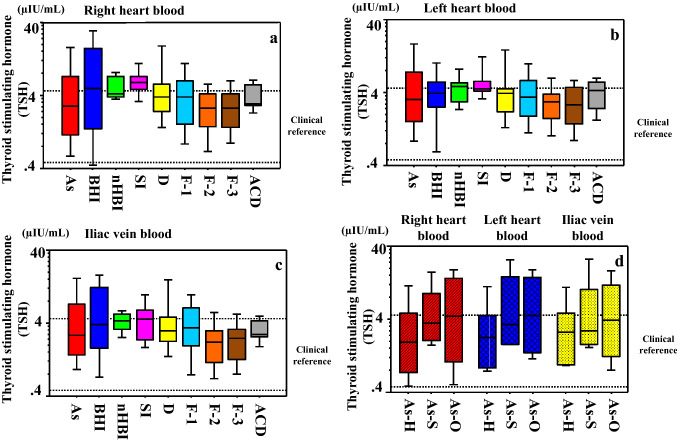
Serum thyroid-stimulating hormone (TSH) concentrations in samples collected from the **a** right cardiac chamber, **b** left cardiac chamber, and **c** iliac vein by cause of death. No significant differences are observed. **d** TSH concentrations by asphyxia subtype (hanging, strangulation, and other). No significant differences were observed. The line bisecting each box represents the median, while the continuous lines outside each box represent the 90% confidence intervals. The area between the two dotted lines in the graph indicates the clinical reference value. Concentrations are log-transformed for graphical presentation only. *As* asphyxia, *BHI* blunt head injury, *nHBI* non-head blunt injury, *SI* sharp instrument injury, *D* drowning, *F-1* fire fatality (CO-Hb < 30%), *F-2* fire fatality (CO-Hb = 30–60%), *F-3* fire fatality (CO-Hb > 60%), *ACD* acute cardiac death

No significant differences in TSH levels were observed between sampling sites by cause of death (Fig. 4a–c) (Table S2). This was also true for subtypes within the asphyxia group (Fig. 4d).

### Histopathology

**Fig. 5:**
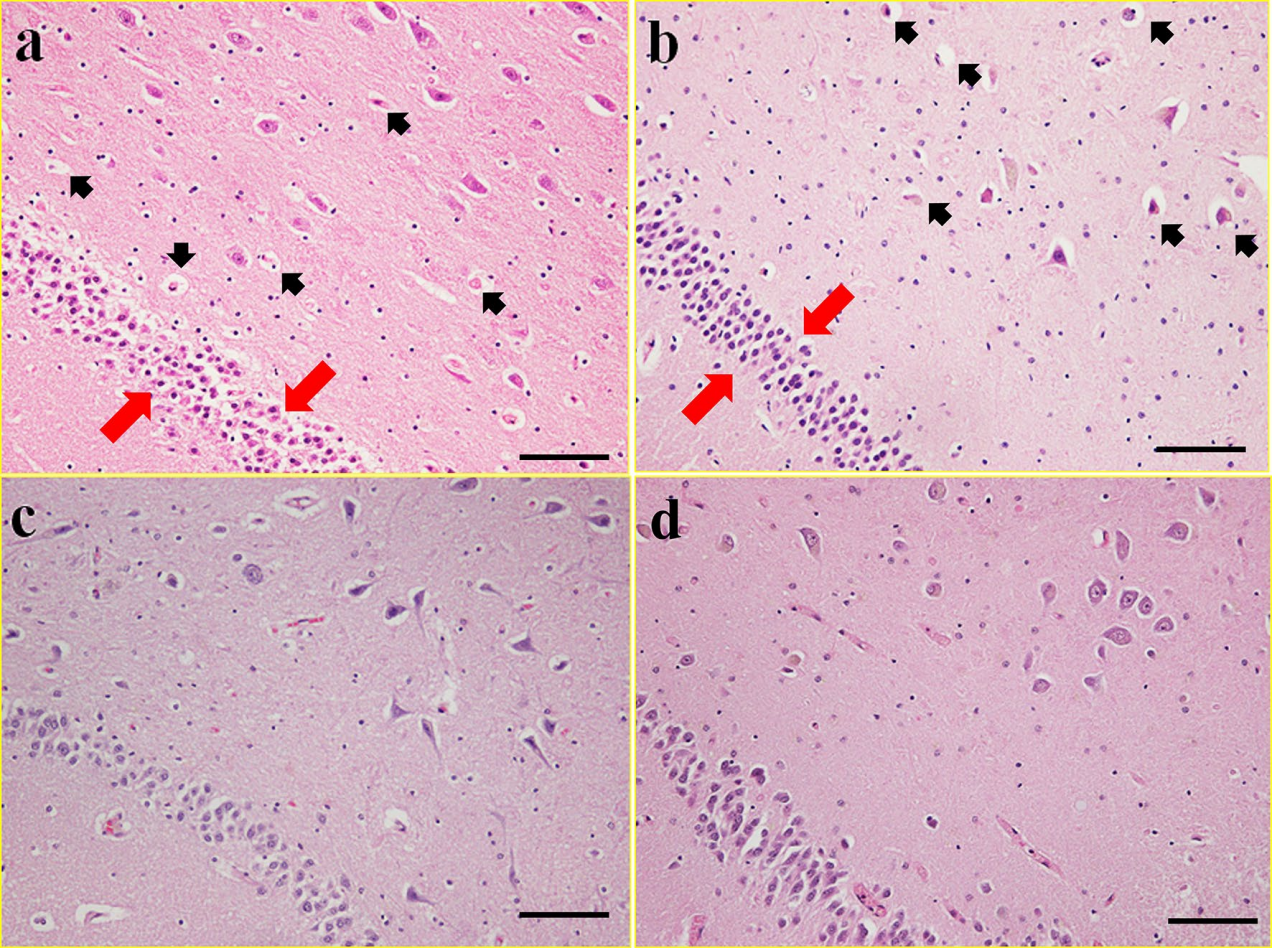
Micropathological findings in the CA4 area of the hippocampus. The black arrow shows neuronal acidophilic changes and falling off. The red arrow shows nuclear condensation: **a** death by hanging in a female in her twenties. **b** Blunt head injury in a male in his 50 s. **c** Sharp instrument injury in a male in his 70 s. **d** Acute cardiac death in a male in his 70 s. All cases were less than 36 h posthumously. Hematoxylin and eosin stain (bar = 50 μm, original magnification × 200)

In cases of asphyxia and blunt head injury with ischemia/hypoxia, findings suggestive of neuronal acidophilic changes, neuronal falling off and nuclear condensation were seen in area CA4 of the hippocampus (Fig. 5).

### Thyroid hormones in HOTHC-sc-4D7 and UD-PTC cell lines

**Fig. 6:**
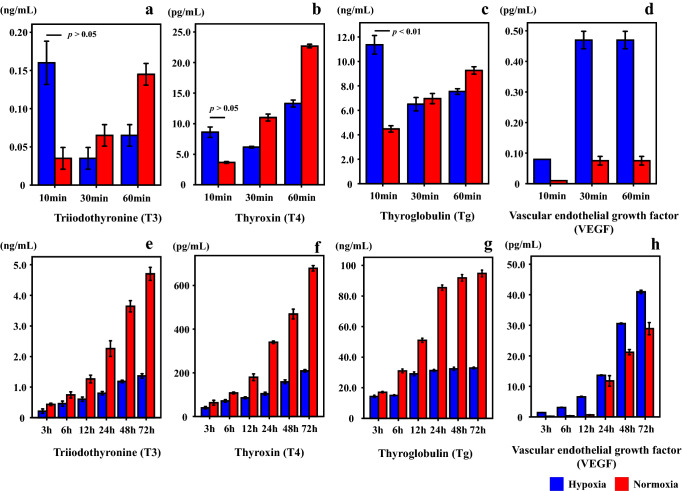
Concentrations of triiodothyronine (T3), thyroxine (T4), thyroglobulin (Tg), and vascular endothelial growth factor (VEGF) in a hormone-secreting HOTHC-sc-4D7 sub-clone derived from human anaplastic thyroid carcinoma (HOTHC) cells cultured under short-term (**a**–**d**) and long-term (**e**–**h**) hypoxic conditions (3% O_2_). After 10 min of culture, T3, T4, and Tg concentrations are found to be higher under hypoxic conditions compared to normoxic conditions (**a**–**c**). However, after > 30 min of culture, these concentrations are lower under hypoxic conditions compared to normoxic conditions (**a**–**c** and **e**–**g**). No decrease in VEGF levels is observed, indicating no loss in cell activity (**d**, **h**)

Immunocytochemistry was used to demonstrate that HOTHC-sc-4D7 cells produced T3 and T4 (Fig. S4). The results of the absorption test showed that the antibody was absorbed with sufficient antigen, but it was not immune-stained (Fig. S5), and this was in agreement with the results of the immunostaining tests (with the exception of the primary antibody shown in Fig. S4c). To confirm the ability of HOTHC-sc-4D7 cells to secrete thyroid-related hormones, the TSH dose was changed (0–30 μIU/ml) and added to the cells, followed by incubation for 3 days. Thereafter, measurement of the T3, T4, and Tg levels showed that they increased with increasing TSH levels (Fig. S6). These results confirmed that, based on the primary antibody targets, T3 and T4, HOTHC-sc-4D7 cells have the ability of secreting thyroid-related hormones. In the cell culture experiment under the hypoxic condition, thyroid-related hormones (T3, T4, and Tg) showed higher concentrations in hormone-secreting HOTHC-sc-4D7 cells cultured under hypoxic conditions (3% O_2_) for 10 min compared to those that were cultured under normoxic conditions (Fig. 6a–c). In hypoxia, the T3 concentration, in particular, exhibited a fivefold increase compared to the normoxia condition. In contrast, the T3, T4, and Tg levels were lower in cells that were cultured under hypoxic conditions for more than 30 min compared to those that were cultured under normoxic conditions (Fig. 6e–g). VEGF levels increased under hypoxic and normoxic conditions and VEGF levels under hypoxic (more than 30 min) conditions were much higher than under normoxic conditions (Fig. 6d, h).

**Fig. 7:**
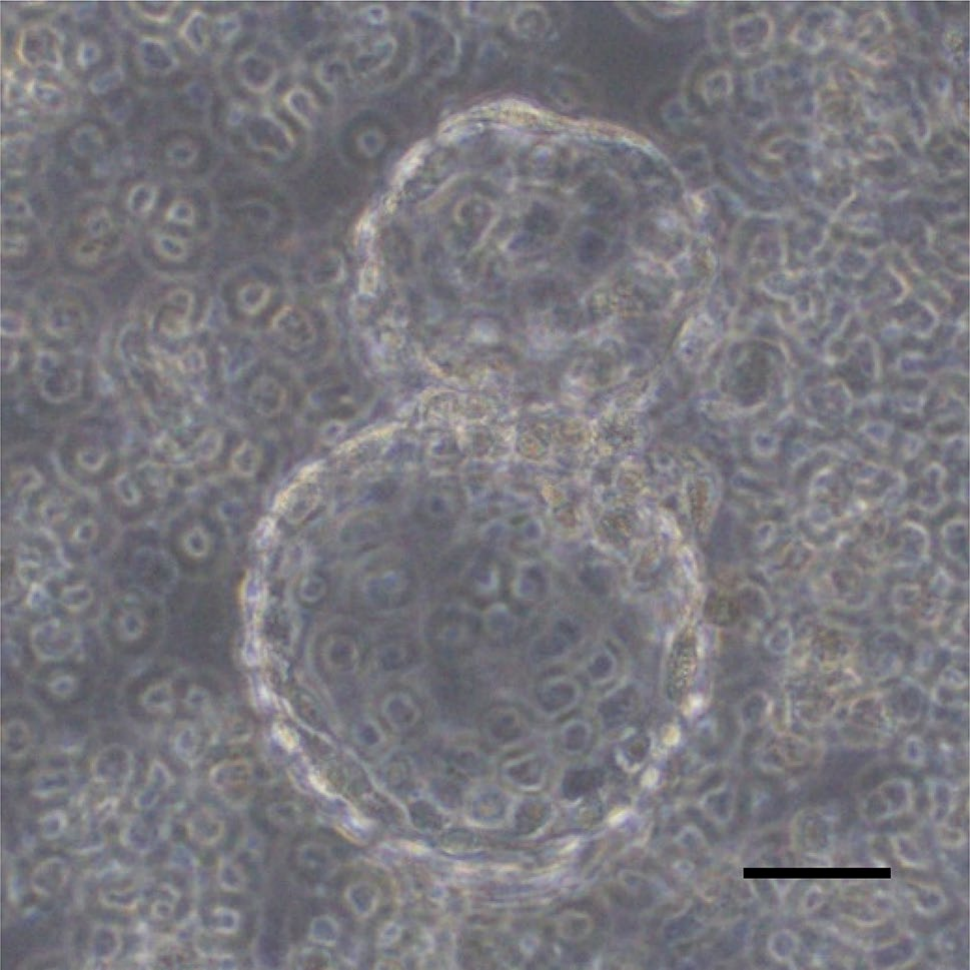
Follicular formation with UD-PTC under phase-contrast microscopy. Bar = 100 μm

**Fig. 8:**
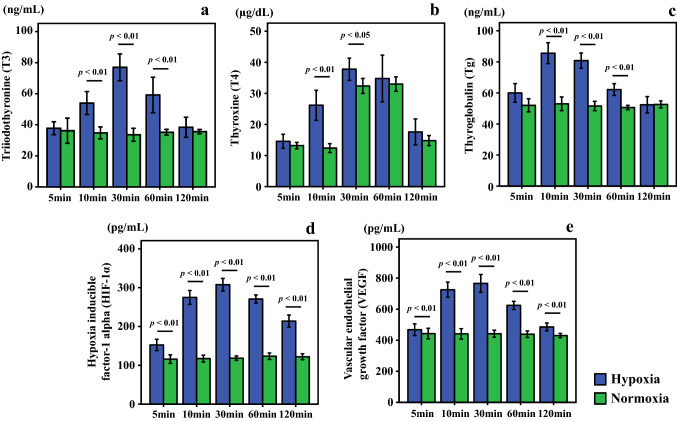
Concentrations of triiodothyronine (T3) (**a**), thyroxine (T4) (**b**), thyroglobulin (Tg) (**c**), hypoxia inducible factor-1 alpha (HIF-1α) (**d**), and vascular endothelial growth factor (VEGF) (**e**) in the UD-PTC cell line derived from human thyroid papillary adenoma cultured under hypoxic conditions (3% O_2_). UD-PTC cells show high levels of T3, T4, and Tg following incubation for 10–30 min under the hypoxic condition and decrease after > 30 to 60 min. HIF-1α levels peak at 30 min after the hypoxic condition. HIF-1α levels under the hypoxic condition are always higher than under the normoxic condition. VEGF levels peak at 30 min after the hypoxic condition and decrease after 60 min

When cultures of UD-PTC cells reached confluency, the monolayer formed balloon structures similar to the thyroid follicular cells (Fig. 7). UD-PTC cells showed higher concentrations of T3 and T4 following incubation for 10–30 min under the hypoxic condition compared to the normoxic condition (Fig. 8a, b). Tg levels peaked at 10 min in the hypoxic condition and decreased after more than 30 min (Fig. 8c). T3 and T4 levels peaked at 30 min after the hypoxic condition and decreased after more than 60 min. To confirm cell function in UD-PTC cells under the hypoxic condition, HIF-1α and VEGF levels were measured. HIF-1α concentrations peaked at 30 min after the hypoxic condition, unlike under the normoxic condition (Fig. 8d, e). HIF-1α levels under the hypoxic condition were always higher than under the normoxic condition. VEGF levels peaked at 30 min after the hypoxic condition and decreased after more than 60 min.

## Discussion

The results of this study showed that the T3 levels in several cases of asphyxia and blunt head injury exceeded the measurement range, although the levels of other thyroid-related hormones remained within the range. These results suggested a high concentration of T3 in blood collected under hypoxic/ischemic conditions. No significant relationships between the levels of TSH and T3, T4, and Tg were observed, suggesting that changes in the levels of T3, T4, and Tg were independent of the levels of TSH.

In the present study, asphyxia and acute/subacute head injuries, representing hypoxic and ischemic conditions of the brain were associated with the highest serum T3 and T4 levels at all sampling sites, with the exception being the T4 levels in samples from the left cardiac chamber. However, no differences in the levels of these hormones were observed between asphyxia subtypes (hanging and strangulation as neck compression, and others), irrespective of the sampling site, suggesting that the T3 and T4 levels increased in response to ischemia/hypoxia. Similarly, asphyxia was associated with a slight but significant increase in serum Tg levels, while the TSH levels did not differ with the cause of death. Acute/subacute brain injury cases showed similar serum T3 and T4 levels as asphyxia cases, suggesting that brain ischemia/hypoxia was related to blunt head injuries. Although cardiogenic and hemorrhagic shock with systemic hypotension are likely to cause secondary cerebral hypoxia/ischemia, this hypoxia/ischemia does not usually cause release of T3 and T4. This could be because the secretion of T3 and T4 in asphyxia and blunt head injury depends on the duration of the survival period, i.e., the period for which the brain experiences hypoxia/ischemia.

Moreover, the serum T3 and T4 levels were not significantly higher in cases of drowning, probably due to the occurrence of pulmonary alveolar destruction rather than ischemia/hypoxia [32]. These results corroborated previous reports where the Tg levels were found to be higher in hanging deaths with neck compression compared to sudden deaths, while the TSH levels tended to remain within the normal range [6, 33, 34]. It has also been shown that mechanical forces applied to the neck region can cause Tg to be released into the circulation, and, therefore, measurements of the Tg levels in bloodstains were undertaken to facilitate the diagnosis of mechanical asphyxia due to neck compression [35, 36]. Similarly, Senol et al. reported that mechanical asphyxia (hanging, manual strangulation, and strangulation by ligature as neck compression) was associated with blood plasma Tg levels > 200 ng/ml (maximum values of 2100 ± 3450 ng/ml), while lower levels (< 200 ng/ml) were associated with other causes of death [7]. In contrast, the Tg levels in the current study did not vary with the cause of death for unknown reasons. Moreover, postmortem serum Tg was secreted in large amounts compared to the clinical serum reference values, regardless of the cause of death.

Clinically, the terminal stage in all patients is respiratory and circulatory failure. Methods of assessing this final condition have not been established in the field of forensic medicine. In the previous stage, that is the survival stage, the levels of various markers fluctuate depending on the prevalent medical condition. There have been many reports on these phenomena. For example, the final stage in drowning might be considered respiratory failure by clinical specialists, but would be considered mainly alveolar damage by forensic specialists [32]. However, the pathology in drowning also depends on the duration of the survival period. Consequently, the pathologies are significantly different in the initial stage of drowning versus that with mechanical asphyxia. Mechanical asphyxia causes systemic hypoxia/ischemia including in the brain. However, acute/sub-acute blunt head injury instead represents a traumatic mechanism that may or may not cause secondary cerebral hypoxia or ischemia, depending on the presence or absence of respiratory depression, intracranial hemorrhage, or cerebral infarction. Moreover, cardiogenic or hemorrhagic shock with systemic hypotension is likely to cause secondary global cerebral hypoxia/ischemia. However, T3 and T4 elevations were observed only in asphyxia and blunt head injury. To investigate the differences between asphyxia and head injury and other causes of death that may involve secondary hypoxic injury, neurohistopathological analyses were conducted. In our previous study, it became clear that hippocampal area CA4 cell injury is caused by cerebral hypoxia accompanied by mechanical asphyxia due to neck compression [37]. As a result, blunt head injury is histopathologically very similar to cases of asphyxia, with nuclear condensation and loss of nerve cells in the hippocampus [38]. These findings were seen with the early symptoms of hypoxia–ischemia encephalopathy and indicated that there was acute hypoxia–ischemia of the brain before death. On the other hand, in both head and non-head trauma, there was more conspicuous loss of nerve cells in groups with long survival periods. In other words, nerve cell loss becomes more pronounced with a prolonged survival period, till ultimately the histopathological findings of hypoxic ischemic encephalopathy become obvious. Based on these histopathological findings, asphyxia and head injury are judged to be conditions with pre-mortem acute hypoxia–ischemia injury and the occurrence of these changes depends on the duration of the survival period. In cases of cardiac disease and death from blood loss, however, no major histopathological changes are seen in the brain. Similarly, no major changes in the blood levels of T3 and T4 are seen. Summarizing these results, both respiratory and circulatory failure are not necessarily seen in all pathologies. Although thyroid hormone levels are not usually evaluated in the final stage, they are believed to change in response to the pathological condition; these changes might become obvious by investigating and comparing the hormone levels with those in the early stage of the survival period.

In addition to these human serum examinations, a basic analysis of changes in thyroid hormone levels in HOTHC-sc-4D7 and UD-PTC cells cultured under hypoxic conditions was performed. A complicated control mechanism exists in the living body. There is an advantage in using a cell culture in that one can simplify and check the several phenomena that happen in the living body. On the other hand, there is also the possibility that the phenomena observed occur only in the cultured cells. Thus, in an attempt to obtain some generalizable conclusion in this study, two kinds of cell lines were used. In HOTHC-sc-4D7 cells, after 10 min of culture, the T3, T4, and Tg levels under hypoxic conditions were higher than those under normoxic conditions, with a fivefold increase being observed in the T3 concentration. Conversely, after 30 min of culture, the T3, T4, and Tg levels under hypoxic conditions were lower than those under normoxic conditions. Furthermore, the levels of VEGF, which is a marker of cell function under hypoxic conditions [39], were measured, and no decreases were observed, suggesting no thyroid cell dysfunction. Secretion of the thyroid hormone decreased under hypoxic conditions compared to normoxic conditions after 30 min of culture, suggesting that this could be a result of long-term hypoxia and decreased cell function. UD-PTC cells showed high levels of T3, T4, and Tg following incubation for 10–30 min under the hypoxic condition, similar to the HOTHC-sc-4D7 cells. In UD-PTC cells that form follicles, T3 and T4 were secreted later than Tg. It was suggested that Tg is secreted at an early stage after hypoxia and is changed to T4 and T3. Furthermore, T3, T4, and Tg levels decreased after 30–60 min under the hypoxic condition, and Tg was no difference between hypoxia and normoxia after 60 min. On the other hand, VEGF levels in UD-PTC cells increase after hypoxia for 30 min, with no difference after 60 min. HIF-1α levels under the hypoxic condition were always higher than under the normoxic condition. These results suggested that the function did not return to the normal condition under hypoxia due to decreased cell function for a long time. In the present UD-PTC cell culture studies, the cell cultures were always exposed to new culture fluid with the circumfusion culture system. With this method, we were able to determine the amount of secretion at every time point. We found that the hormone secretory capacity of the cultured cells increased after 10–30 min of hypoxia and higher blood hormone levels were maintained even in samples collected much after death. On the other hand, in our study, hormone levels remained stable for 72 h in the human samples.

Cultured cell experiments showed that T3, T4, and Tg are secreted under hypoxia for a short period in both HOTHC-sc-4D7 cells and UD-PTC cells. These results support the increase of T3 and T4 seen in hypoxia/ischemia in human death cases. These findings suggest that post-mortem increases in the T3, T4, and Tg levels under hypoxic conditions occurred independent of TSH control. In the experiment using the HOTHC cell line, thyroid hormones were released within 10 min of death in acute death cases, this increase in thyroid hormone levels due to acute ischemia/hypoxia in human cases being supported by the results of the cell culture experiment. Subsequent increases in thyroid hormone are thought to indicate a long circulation failure condition during survival period. Since HOTHC-sc-4D7 cells do not form follicles, they secrete Tg without incorporating it into follicles, but UD-PTC cells form follicles and secrete Tg into follicles. HOTHC-sc-4D7 cells do not have follicles, so that it is expected that Tg was converted to T4 and T3 immediately. In UD-PTC, Tg was stored in follicles and converted to T4 and T3 from follicular cells and secreted, so it is considered that there was a time lag in the secretion of each, since cells were formed into follicles. In addition, the difference between T3 and T4 secretion in HOTHC-sc-4D7 cells may also have been related to UD-PTC cells being more resistant to hypoxia than HOTHC-sc-4D7 cells and Tg not being suppressed in UD-PTC cells. Tg was secreted under the hypoxic condition in cultured cells, but human autopsy cases did not show an increase under the hypoxic condition perhaps because a large amount of Tg already exists in the body and the short half-life of Tg in the acute hypoxic condition.

In conclusion, although death from asphyxia as the hypoxia/ischemia condition was associated with higher levels of thyroid hormones, these levels did not differ with the presence or absence of neck compression, suggesting that the observed increases in T3 and T4 could be attributed to ischemia/hypoxia rather than mechanical stimulation of the neck. In contrast, in autopsy cases, serum Tg and TSH levels did not differ significantly with the cause of death, indicating a lack of hypothalamic regulation. These results suggest that post-mortem measurements of thyroid function can be used to facilitate the diagnosis of systemic ischemia/hypoxia, but these results cannot be used to confirm or rule out physical neck compression. Therefore, an increase in the levels of thyroid-related hormones does not always indicate neck compression. However, biochemical analysis using human blood and cultured cell experiments suggests that T3, T4, and Tg levels increase under hypoxic conditions independent of TSH control. These results suggest the usefulness of the thyroid-related hormones as new markers for the diagnosis of hypoxia.

## Electronic supplementary material

Below is the link to the electronic supplementary material.

Supplementary file 1 (PDF 471 kb)
