# Supplementary material for: Thyroid-related hormones as potential markers of hypoxia/ischemia
Source: Hum Cell. 2020 Mar 7;33(3):545–58. doi: 10.1007/s13577-020-00341-x (PMC7324426; doi:10.1007/s13577-020-00341-x)
Supplement: Supplementary file 1 — (PDF 471 kb) [file 13577_2020_341_MOESM1_ESM.pdf]

Thyroid-related hormones as potential markers of hypoxia/ischemia

Human Cell

Naoto Tani [1,2*], Mayumi Ishikawa [3], Miho Watanabe [1,4], Tomoya Ikeda [1,2], Takaki Ishikawa [1,2]

[1] Department of Legal Medicine, Osaka City University Medical School, Asahi-machi 1-4-3, Abeno, Osaka 545-8585, Japan

[2] Forensic Autopsy Section, Medico-legal Consultation and Postmortem Investigation Support Center, c/o Department of Legal Medicine, Osaka City University Medical School, Asahi-machi 1-4-3, Abeno, Osaka 545-8585, Japan

[3] Center of Endocrinology, Diabetes and Arteriosclerosis, Nippon Medical School Musashikosugi Hospital, Kosugi-cho 1-396, Nakahara-ku, Kawasaki 211-8533, Japan

[4] Laboratory of Clinical Regenerative Medicine Department of Neurosurgery, Faculty of Medicine, University of Tsukuba, Laboratory of Advanced Research D326 1-1-1, Tennodai, Tsukuba-City, Ibaraki 305-8575, Japan

*Corresponding author: Naoto Tani

Department of Legal Medicine, Osaka City University Medical School, Asahi-machi 1-4-3, Abeno, 545-8585 Osaka, Japan

Tel.: 81-6-6645-3767; fax: 81-6-6634-3871

E-mail address: tani.naoto@med.osaka-cu.ac.jp

Fig. S1. Correlations of concentrations of thyroid-related hormones in blood samples from the right cardiac chamber.

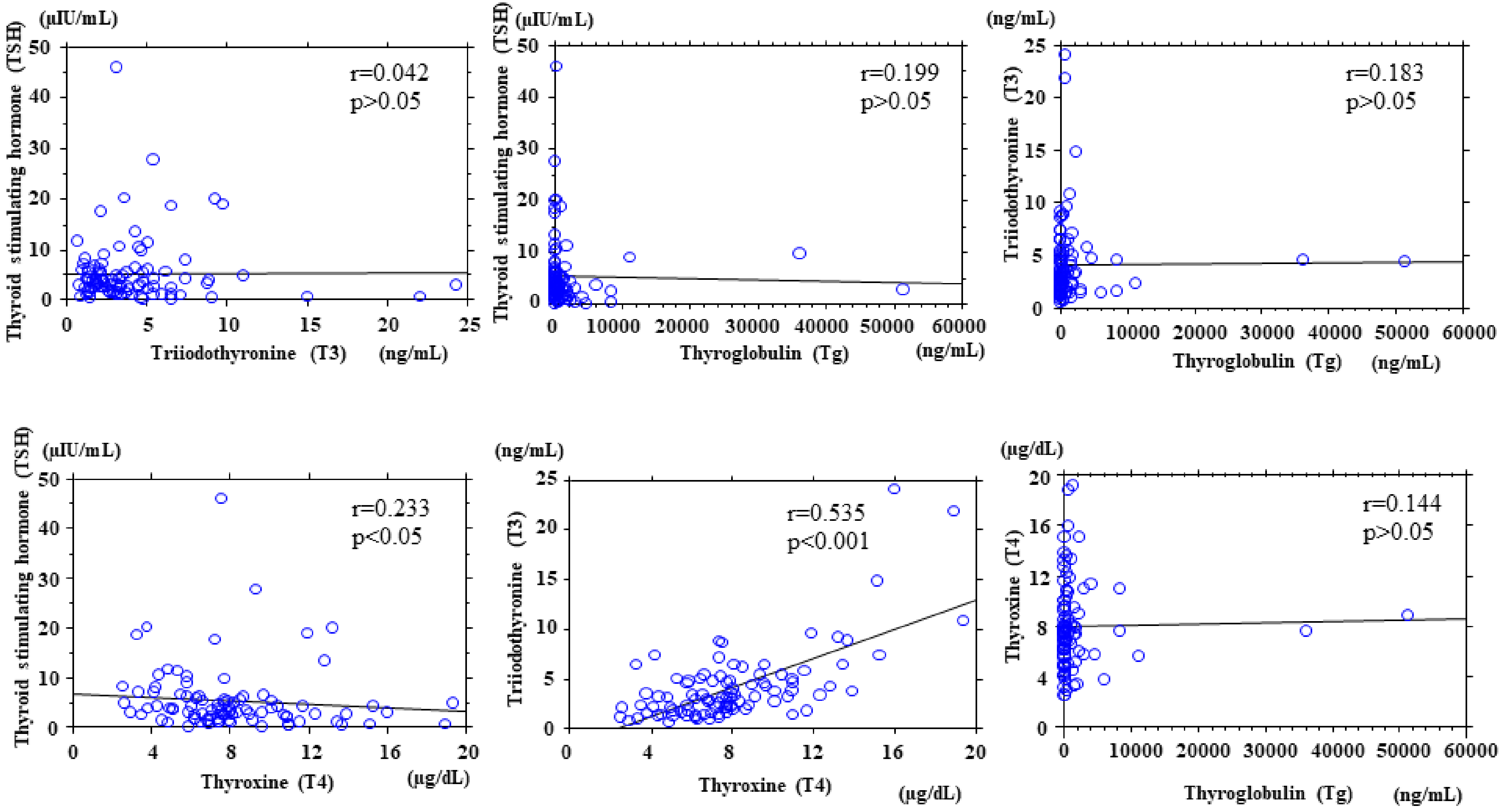

Fig. S2. Correlations of concentrations of thyroid-related hormones in blood samples from the left cardiac chamber.

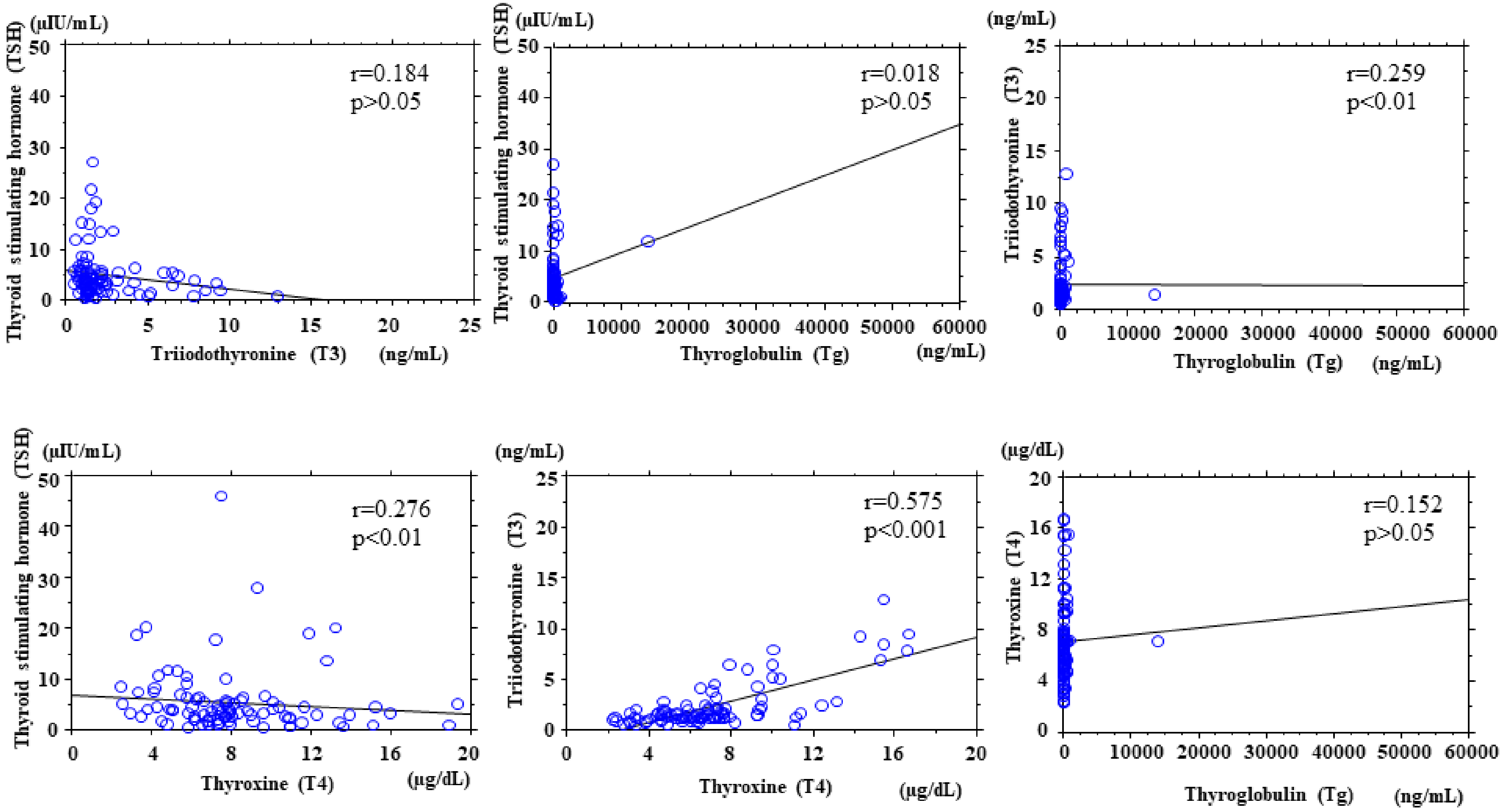

Fig. S3. Correlations of concentrations of thyroid-related hormones in blood samples from the iliac vein.

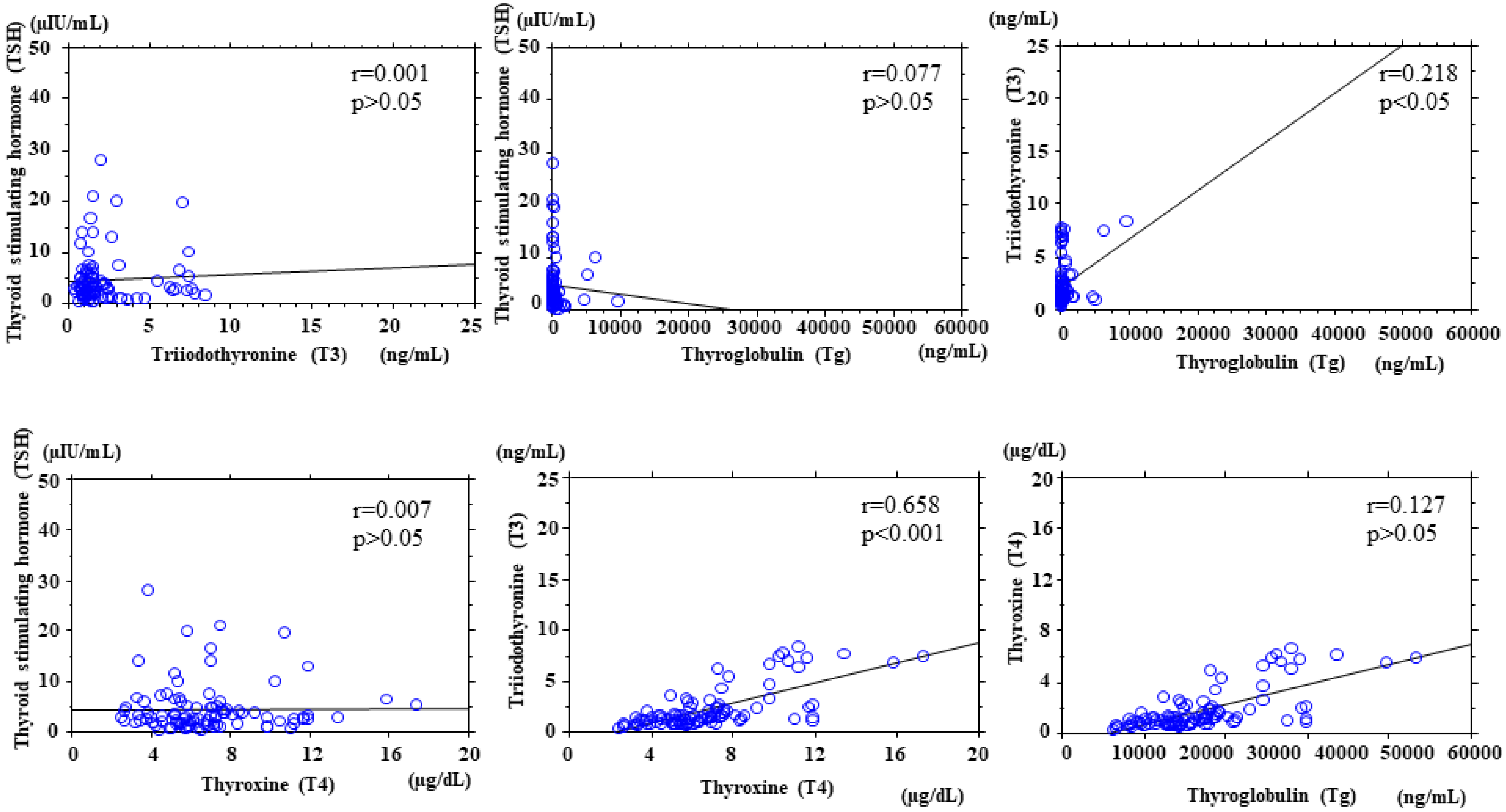

Fig. S4. Immunocytochemical analysis of a hormone-secreting HOTHC-sc-4D7 sub-clone derived from a human anaplastic thyroid carcinoma (HOTHC) cell line. Fluorescence immunostaining studies for triiodothyronine (T3; a-i: positive staining with Alexa Fluor 488, a-ii: DAPI-stained nuclei, a-iii: combined Alexa Fluor 488 and DAPI staining), thyroxine (T4; b-i: positive staining with Alexa Fluor 488, b-ii: DAPI-stained nuclei, b-iii: combined Alexa Fluor 488 and DAPI staining). HOTHC-sc-4D7 cells were stained without primary antibodies against T3 and T4 (c-i: negative staining with Alexa Fluor 488, c-ii: DAPI-stained nuclei I, c-iii: combined with Alexa Fluor 488 and DAPI staining). Bar = 20 μm.

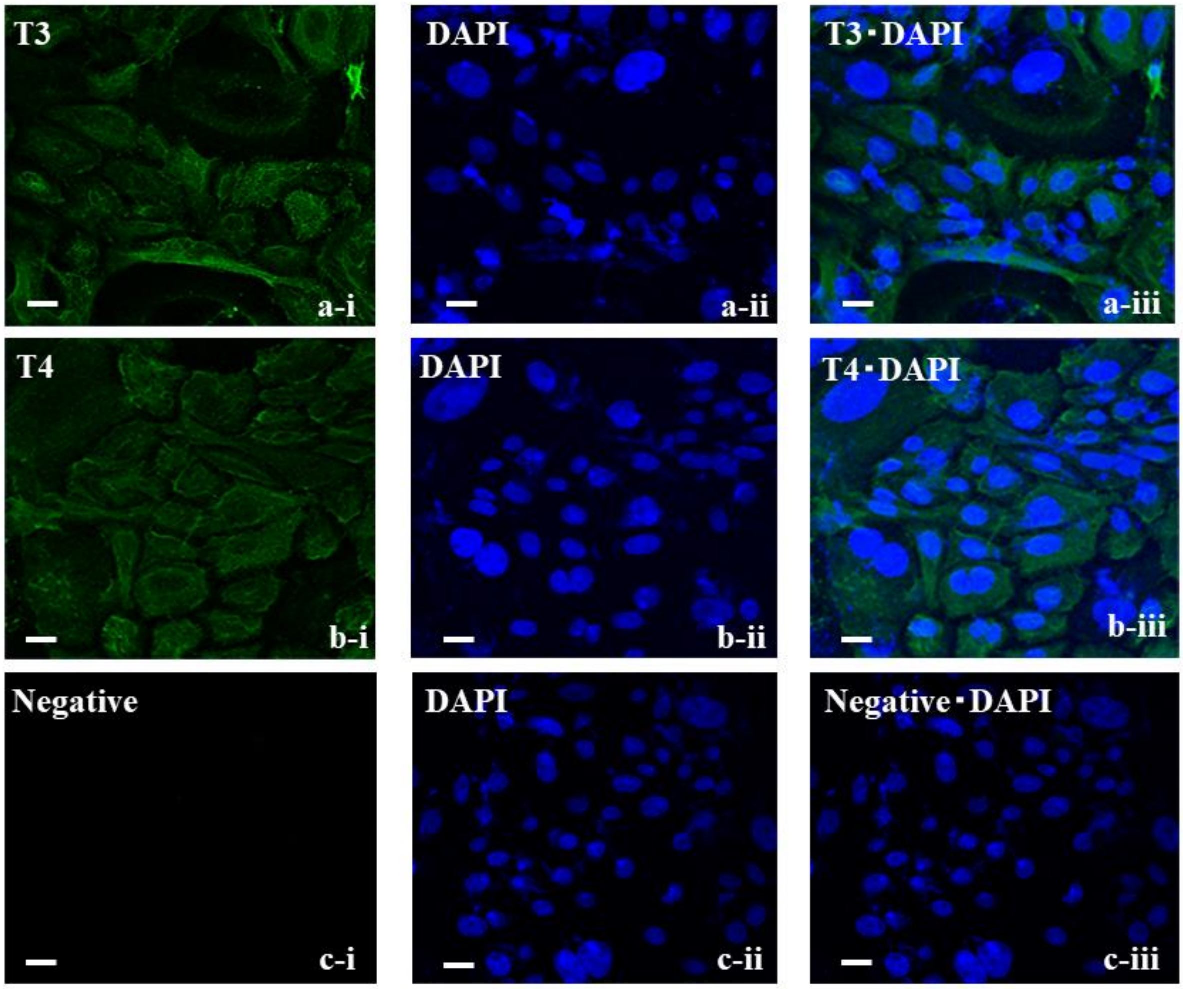

Fig. S5. Immunostaining of HOTHC-sc-4D7 using an antibody targeted against absorbed T3 (a) and T4 (b). (c) Negative control excluding the antibody and DAPI.

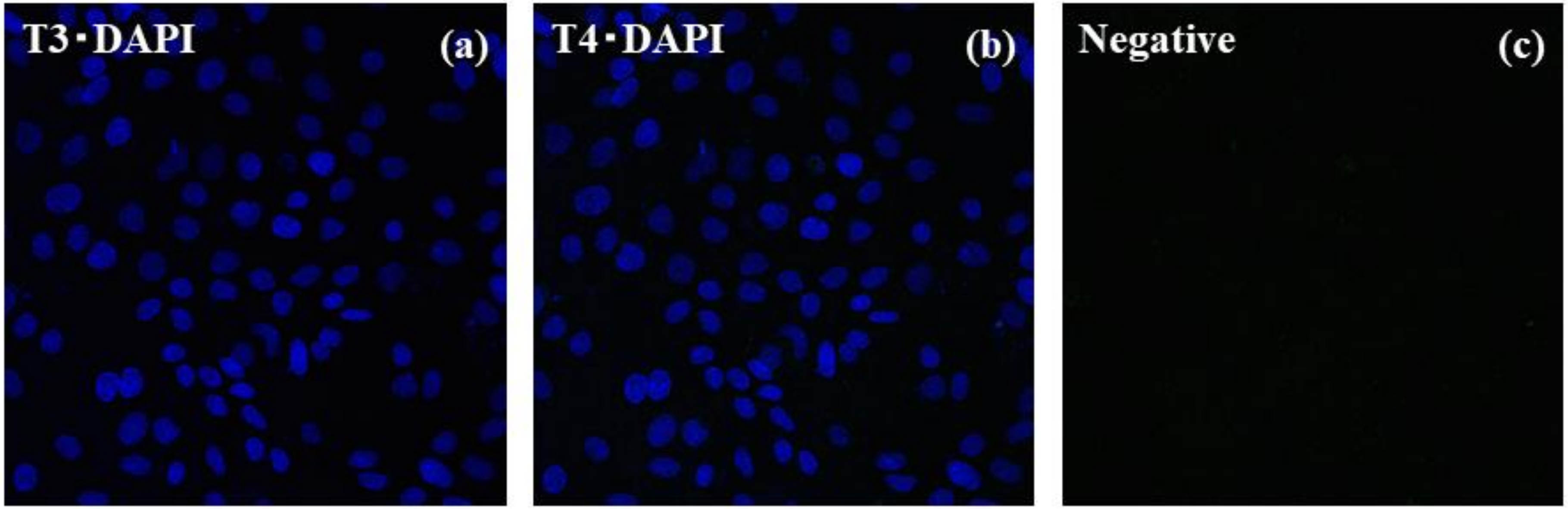

Fig. S6. These graphs confirm the ability of a hormone-secreting HOTHC-sc-4D7 sub-clone derived from human anaplastic thyroid carcinoma (HOTHC) cells to secrete thyroid-related hormones. The thyroid-stimulating hormone (TSH) dose was changed (0–30 µIU/ml) and added to HOTHC-sc-4D7 cells, followed by incubation for 3 days. Thereafter, the triiodothyronine (T3) (a), thyroxine (T4) (b), and thyroglobulin (Tg) (c) levels were measured. A TSH concentration of "0 µIU/ml" indicates Dulbecco's Modified Eagle Medium: Nutrient Mixture F-12 containing only 5% fetal bovine serum. The results show that the T3, T4, and Tg levels increased along with the TSH level.

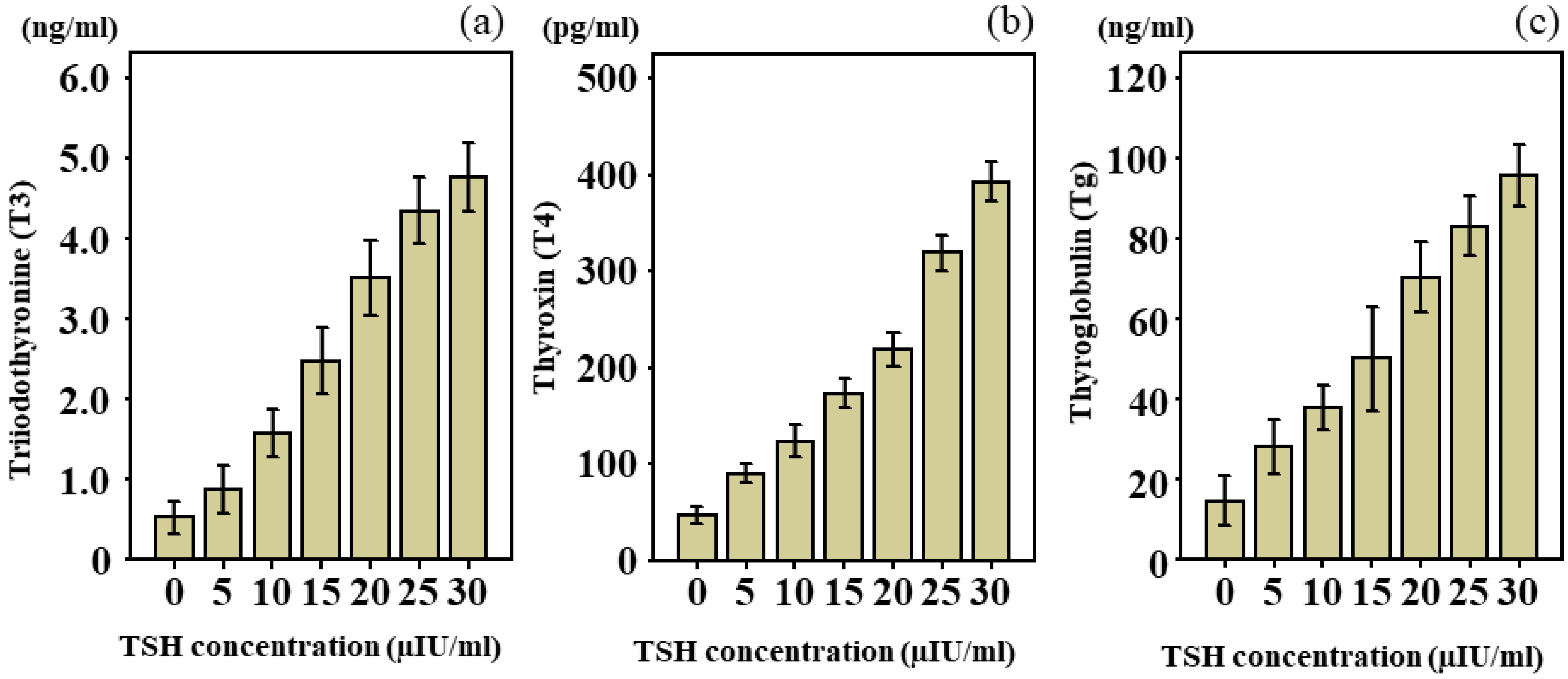

Table S1. Triiodothyronine (T3) and thyroxin (T4) concentrations in blood samples collected from the right and left cardiac chambers and iliac vein.

Cause of death	Triiodothyronine (T3) (ng/mL)			Thyroxin (T4) (μg/dL)		
	Cardiac blood left (median)	Cardiac blood right (median)	Iliac vein blood (median)	Cardiac blood left (median)	Cardiac blood right (median)	Iliac vein blood (median)
Asphyxia						
Hanging	0.55-12.9 (5.79)	1.74-22.0 (6.20)	1.15-7.30 (3.74)	4.60-16.7 (12.1)	5.47-18.9 (11.9)	5.17-11.9 (10.5)
Strangulation	0.93-9.24 (2.90)	2.32-24.2 (5.20)	0.94-7.68 (2.25)	3.07-15.4 (6.65)	3.23-16.0 (7.39)	3.83-13.4 (8.69)
Other*	1.18-5.21 (2.13)	2.09-9.70 (5.45)	1.19-8.38 (2.29)	5.07-9.99 (7.31)	7.23-13.4 (10.4)	5.67-11.2 (7.10)
Blunt injury						
Head injury						
Acute	1.07-2.13 (1.48)	2.09-9.20 (3.55)	0.84-6.29 (1.52)	2.24-7.67 (6.15)	3.72-13.2 (7.48)	3.34-7.25 (5.78)
Subacute	1.85-6.50 (2.26)	3.16-11.0 (6.90)	1.50-6.60 (1.85)	4.80-10.0 (8.20)	5.82-19.3 (10.7)	4.39-9.80 (7.50)
Non-head injury						
Acute	0.94-1.70 (1.26)	1.26-2.35 (2.19)	0.75-1.41 (1.11)	3.33-7.57 (3.90)	4.10-8.84 (5.10)	3.61-7.26 (5.16)
Subacute	0.84-1.52 (0.94)	1.17-2.13 (1.25)	0.61-1.29 (0.81)	2.45-4.57 (4.83)	2.51-4.92 (4.29)	2.69-5.60 (3.32)
Sharp instrument injury	0.69-2.06 (1.18)	1.02-5.02 (1.96)	0.54-1.13 (0.96)	2.32-6.07 (4.98)	2.57-7.71 (5.52)	2.63-6.28 (4.26)
Drowning	0.77-7.90 (1.52)	1.39-5.42 (2.89)	0.77-7.90 (1.52)	3.04-10.1 (6.03)	3.47-10.1 (6.86)	3.40-7.70 (6.68)
Fire fatalities						
CO-Hb < 30%	0.60-3.09 (1.24)	0.67-4.53 (2.91)	0.69-7.80 (1.29)	4.75-9.48 (6.41)	4.89-9.58 (6.71)	4.80-15.8 (5.76)
CO-Hb = 30-60%	0.81-3.86 (1.35)	0.94-4.47 (2.28)	0.81-7.40 (1.38)	4.70-12.4 (7.11)	4.80-13.9 (7.54)	4.54-17.3 (5.83)
CO-Hb > 60%	0.87-6.90 (1.39)	1.79-7.40 (3.44)	0.75-2.71 (1.48)	3.47-15.3 (7.27)	4.19-15.2 (7.94)	3.26-8.30 (6.46)
Cardiac dysfunction	0.55-6.00 (1.99)	0.81-6.20 (4.32)	0.42-5.50 (1.27)	3.41-9.31 (5.63)	2.91-9.67 (7.73)	2.46-7.80 (4.87)
Total	0.55-12.9 (1.51)	0.67-24.2 (3.21)	0.42-8.38 (1.43)	2.24-16.7 (6.59)	2.51-19.3 (7.48)	2.46-17.3 (6.20)

*Other; choking (n = 3), traumatic asphyxia (n = 3), smothering (n = 1)

Table S2. Thyroglobulin (Tg) and thyroid stimulating hormone (TSH) concentrations in blood samples collected from the right and left cardiac chambers and iliac vein.

Cause of death	Thyroglobulin (Tg) (ng/ml)			Thyroid stimulating hormone (TSH) (μIU/ml)		
	Cardiac blood left (median)	Cardiac blood right (median)	Iliac vein blood (median)	Cardiac blood left (median)	Cardiac blood right (median)	Iliac vein blood (median)
Asphyxia						
Hanging	8.37-761 (35.3)	15.9-4040 (267)	8.34-1710 (319)	0.77-13.6 (2.27)	0.385-13.6 (1.95)	0.913-13.0 (2.64)
Strangulation	22.5-636 (179)	63.3-11000 (533)	19.1-6340 (220)	1.80-27.1 (3.39)	1.76-18.7 (3.52)	1.61-28.2 (2.79)
Other*	12.9-1120 (158)	32.1-8380 (846)	9.12-9540 (236)	1.13-19.3 (4.51)	0.392-19.1 (4.42)	0.723-19.9 (3.93)
Blunt injury						
Head injury						
Acute	13.0-193 (20.6)	13.5-1450 (168)	14.7-368 (91.9)	0.804-14.8 (3.89)	1.01-46.0 (10.6)	1.00-20.0 (7.26)
Subacute	33-348 (49.1)	36.4-4660 (792)	38.5-500 (124)	0.352-5.47 (4.28)	0.227-5.00 (2.79)	0.32-5.88 (3.26)
Non-head injury						
Acute	3.96-17.9 (15.3)	8.11-612 (224)	5.22-329 (27.7)	2.28-5.44 (3.03)	3.55-7.30 (3.84)	3.29-5.97 (5.28)
Subacute	24.5-212 (37.5)	56.0-239 (114)	12.6-250 (40.8)	4.57-8.65 (5.20)	3.88-8.38 (4.53)	2.48-5.05 (3.50)
Sharp instrument injury	0.32-663 (59.0)	19.4-2820 (1206)	10.3-430 (61.1)	3.20-13.2 (4.55)	3.12-11.5 (6.00)	1.80-10.1 (4.47)
Drowning	13.2-108 (33.1)	11.2-6120 (234)	7.59-413 (57.0)	1.24-21.5 (3.89)	1.46-27.8 (3.82)	1.29-21.2 (3.14)
Fire fatalities						
CO-Hb < 30%	1.97-593 (13.8)	6.30-2380 (80.1)	1.70-860 (50.0)	1.05-11.7 (3.50)	0.802-11.6 (3.77)	0.599-11.6 (3.46)
CO-Hb = 30-60%	2.63-13900 (27.5)	4.46-36100 (30.4)	1.80-4950 (22.0)	0.801-12.0 (3.04)	0.081-9.96 (2.65)	0.073-6.69 (2.18)
CO-Hb > 60%	9.43-360 (35.1)	11.6-1990 (63.4)	8.52-786 (44.7)	0.355-7.04 (2.70)	0.08-8.05 (2.63)	0.196-6.74 (2.49)
Cardiac dysfunction	7.82-84.8 (33.1)	182-51200 (696)	32.8-227 (43.4)	1.59-6.35 (4.28)	2.25-6.60 (3.10)	1.84-5.00 (2.82)
Total	0.32-13900 (34.8)	4.46-51200 (230)	1.70-9540 (48.8)	0.35-27.1(3.46)	0.08-46.0 (3.45)	0.07-28.2 (2.85)

*Other; choking (n = 3), traumatic asphyxia (n = 3), smothering (n = 1)
